# A Concept for MRI-Based Cholesteatoma Detection in Cochlear Implant Recipients

**DOI:** 10.3390/audiolres15060162

**Published:** 2025-11-21

**Authors:** Lukas Woltersdorf, Rayoung Kim, Alexander Rempen, Christoph Pfeiffer, Lars-Uwe Scholtz, Christiane Schimmack, Daniela Eickenjäger, Rüdiger Steinbach, Ingo Todt

**Affiliations:** 1 Department of Otolaryngology, Head and Neck Surgery, Medical School OWL, Bielefeld University, Campus Mitte, Klinikum, 33604 Bielefeld, Germany; lukas.woltersdorf@klinikumbielefeld.de (L.W.); rayoung.kim@klinikumbielefeld.de (R.K.); alexander.rempen@klinikumbielefeld.de (A.R.); christoph.pfeiffer@klinikumbielefeld.de (C.P.); lars-uwe.scholtz@klinikumbielefeld.de (L.-U.S.); 2 Diranuk, Radiology Center, 33602 Bielefeld, Germany; christiane.schimmack@lifelink-medical.com (C.S.); daniela.eickenjaeger@lifelink-medical.com (D.E.); ruediger.steinbach@lifelink-medical.com (R.S.); 3 Alexianer St. Gertrauden-Krankenhaus, Paretzerstr. 12, 10713 Berlin, Germany

**Keywords:** CI, MRI, cholesteatoma

## Abstract

Introduction: Cochlear implantation is the treatment of choice for severe hearing loss and deafness. Cholesteatomas can cause this deafness. A frequently used procedure in the course of surgical rehabilitation is a subtotal petrosectomy combined with a cochlear implant. The clinical follow-up of residual cholesteatomas is related to the blind sac closure difficult. Cholesteatoma MRI sequence-related CI magnet artefacts make follow-up challenging. Recent developments in combining cochlear implants and necessary MRI examinations enable the assessment of the internal auditory canal and cochlea. The study aimed to develop a procedure for detecting cholesteatomas in patients with cochlear implants using magnetic resonance imaging (MRI). Methods: Ex vivo MRI examinations were performed on five volunteers with fixed cochlear implants (Medel Synchrony) and swim caps. MRI examinations were performed at 1.5 T and 3 T using EPI, HASTE, and RESOLVE sequences (Siemens). The position of the implant was 12 cm distal to the external auditory canal, with anteversional head position of the volunteers in the MRI. Results: Due to artefact effects, assessment of the ipsilateral and contralateral mastoid is not possible with EPI sequences and a cochlear implant. The combination of cholesteatoma-detecting MARS sequences (HASTE, RESOLVE), a distal implant position, and a specific head position allows the assessment of the ipsilateral mastoid. Conclusions: Postoperative cholesteatoma assessment after CI implantation and subtotal petrosectomy appears to be possible under 1.5 T and 3 T, considering the MRI sequence, implant position, and head position.

## 1. Introduction

Given the importance of MRI as a routine diagnostic tool in many clinical pathways, the likelihood of needing a scan at some point in a lifetime is high. This finding holds even for cochlear implant recipients [1].

Developments of the CI magnet to bipolar diametral magnets, which align to the applied magnetic field, allow a painless procedure without complications [2]. The position of the implant at the head [3], the position of the head inside the MRI scanner (chin to chest) [4], and a proper sequence allow the routine assessment of the internal auditory canal and the cochlea.

These findings allow the routine follow-up of vestibular schwannoma and intralabyrinthine schwannoma in combination with a cochlear implant [5,6]. Further development and application of MARS sequences enable the reduction in artifacts around the implant and improved visualization of central structures [7,8,9].

**Figure 1:**
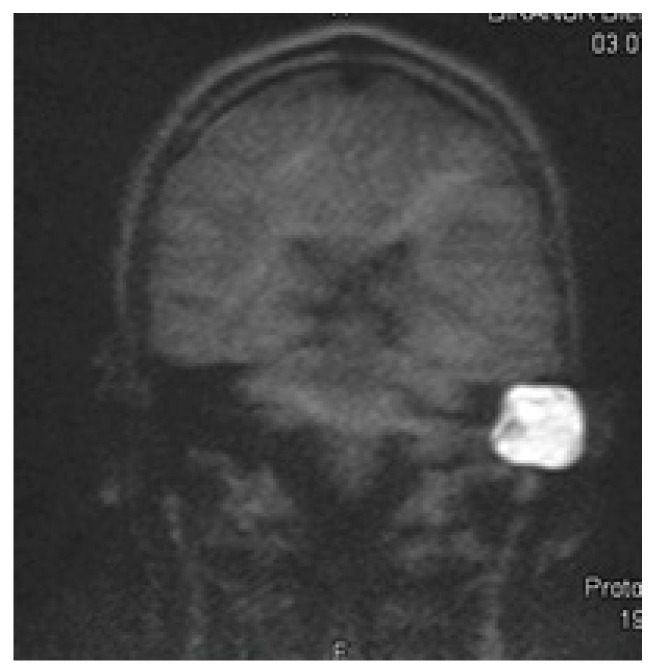
Exemplary cholesteatoma of a left mastoid cavity scan with HASTE at 1.5 T.

Cholesteatoma surgery makes a clinical, in most cases, surgical follow-up mandatory. The introduction of specific MRI sequences into the field of cholesteatoma detection allowed a non-surgical radiological second look [10] for the first time. Besides the initial single-shot echo planar imaging (EPI) DWI detection sequence [11], non-EPI echo planar imaging DWI was developed, achieving higher sensitivity and specificity of 91% and 97%, respectively [12,13]. The disadvantage of most cholesteatoma detection sequences is their high susceptibility to magnetic fields. Therefore, significant distortive artifacts can be generated by an implant [14], which makes the evaluation of cholesteatoma impossible. Although modified cholesteatoma sequences, such as multishot EPI DWI RESOLVE and single-shot non-epi DWI HASTE (Figure 1), are assumed to be less susceptible to magnets with acceptable rates of cholesteatoma detection [15]

Subtotal petrosectomy (SP) contains the complete exenteration of pneumatized cells of the mastoid, removal of epithelialized cells, and removal of the posterior wall. Closure of the eustachian tube and a blind sac closure of the EAC is performed after filling the cavity with fat. This technique, developed in the late 1950s [16], was combined with a cochlear implant in the late 1990s [17] as a surgical solution for many pathologies. It is described as being performed in cases of COM, cholesteatoma, temporal bone fractures, otosclerosis, vestibular schwannomas, and inner ear malformations, in combination with a cochlear implant to create a dry cavity [18,19]. Related to the assumed good safety profile, the SP is widely used. Caused by the closure of the blind sac, a follow-up of the underlying etiological disease is so far based on clinical observation and usually involves a CT scan. Since CT is radiologically per se not able to detect residual cholesteatoma, there is a so far unsolved follow-up gap. As previously described, MRI is a well-established radiological tool for the follow-up of cholesteatoma.

Different authors have described the rate of cholesteatoma in the groups of PS and CI as ranging from 9.3% to 20% [20,21,22]. It can be assumed that the rate of undetected cholesteatoma is different. Recently, Macielak et al. described the rate of cholesteatoma in patients with recurrent disease after surgery (SP) as 80%.

This study aimed to develop a procedure for detecting cholesteatomas in patients with cochlear implants using magnetic resonance imaging (MRI).

## 2. Method

We performed MRI scans on five volunteers (three males, two females, mean age of 42, regular head anatomy) with a cochlear implant (Synchrony 2, MED-EL, Innsbruck, Austria). The implants were fixed with a swimming cap at a 120° angle from the nasion to the external ear canal line, with a distance of 12 cm for all volunteers. Inside the scanner, the volunteers were advised to scan in a chin-to-chest (anteversion) position [4]. A cushion supported the head. Each scan was performed with three volunteers.

1.5 T scanning was performed with a MAGNETOM Altea scanner (Siemens, Erlangen, Germany). Using a MAGNETOM Skyra scanner (Siemens, Erlangen, Germany), 3T scanning was performed. The performed sequences are shown in Table 1.

SAR values were calculated for each sequence. Related to the sequence installed on the different scanners, sequences could not be performed in all scanners.

The ethical board of the Westfälische Wilhelms Universität (2019, 135 fS, 20 August 2019) gave its approval for the study.

## 3. Results

### 3.1. EP2D-Diff-Sequence

In all three subjects, the EP2D diffusion sequence shows a pronounced susceptibility artifact in both the axial and coronal planes with areal signal loss and, in some cases, considerable geometric distortions. Around the cochlear implant, central hypointense signal losses with hyperintense fringes are impressive, whereby these fringe phenomena appear particularly pronounced in the coronal plane.

**Figure 2:**
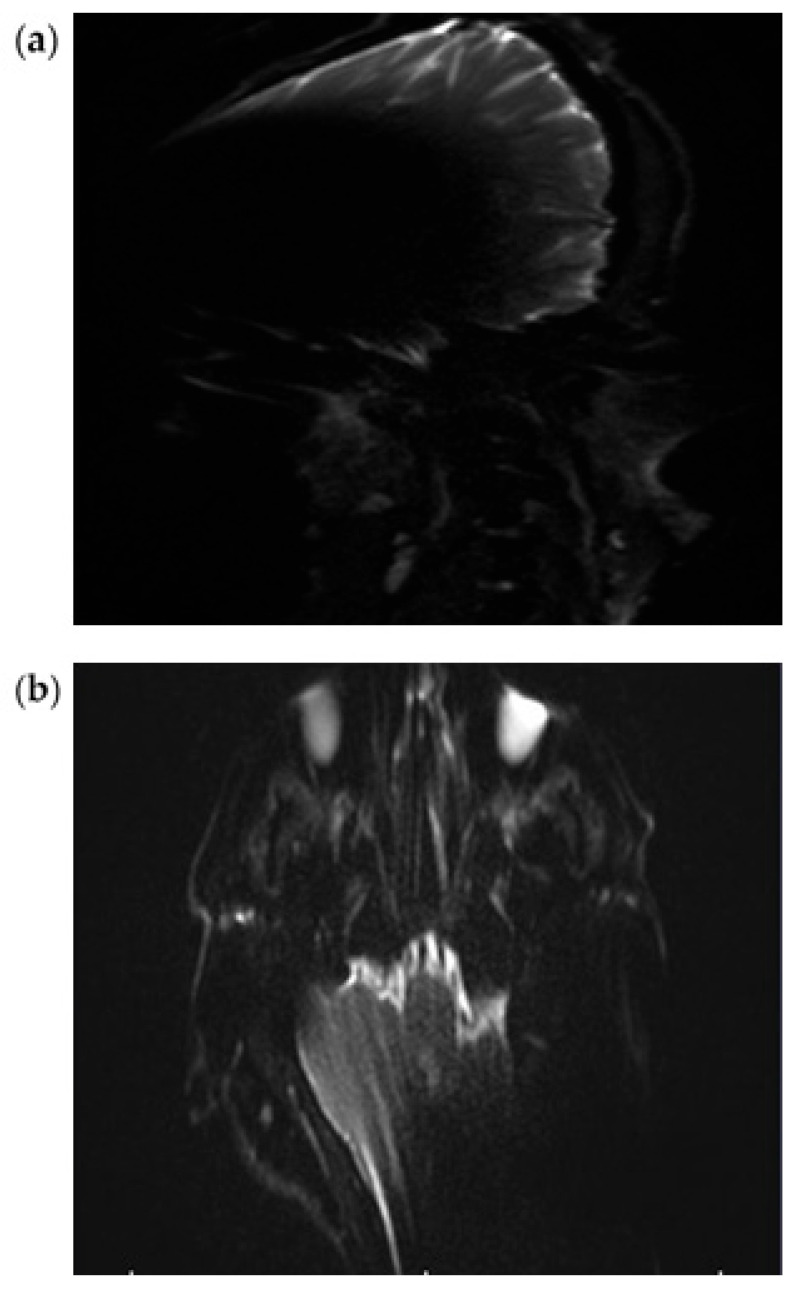
Exemplary scan with an EPI2D sequence at 3T in a coronal (**a**) and axial plane (**b**).

Due to the pronounced stretching and distortion effects, anatomically correct visualization of the mastoid cavities, the external auditory canal, and the internal auditory canal is considerably impaired on both sides (Figure 2a,b).

### 3.2. RESOLVE Sequence

In the transverse as well as in the coronal image plane, a localized signal loss in the region of the cochlear implant can be seen, corresponding to a pronounced metal-induced susceptibility artifact. The average artifact extension in the axial orientation is 108 mm (subject 1: 103 mm; subject 2: 106 mm; subject 3: 116 mm), while in the coronal plane, it is 146 mm (subject 1: 156 mm; subject 2: 147 mm; subject 3: 134 mm). In the coronal image, a disproportionate craniocaudal and mediolateral artifact extension is also impressive, which extends beyond the temporal lobe to the midline and projects into deeper contralateral brain sections.

In each case, the artifacts are accompanied by geometric distortions as well as grid- or band-like signal inhomogeneities in the peripheral area.

In all three cases examined, the ipsilateral mastoid air cell can be visualized mainly in both planes. In subjects 2 and 3, however, the distal section of the ipsilateral mastoid exhibits a partial artifact overlay, which limits assessment. The contralateral mastoid cavity can be assessed in its full extent in all subjects without restrictions.

**Figure 3:**
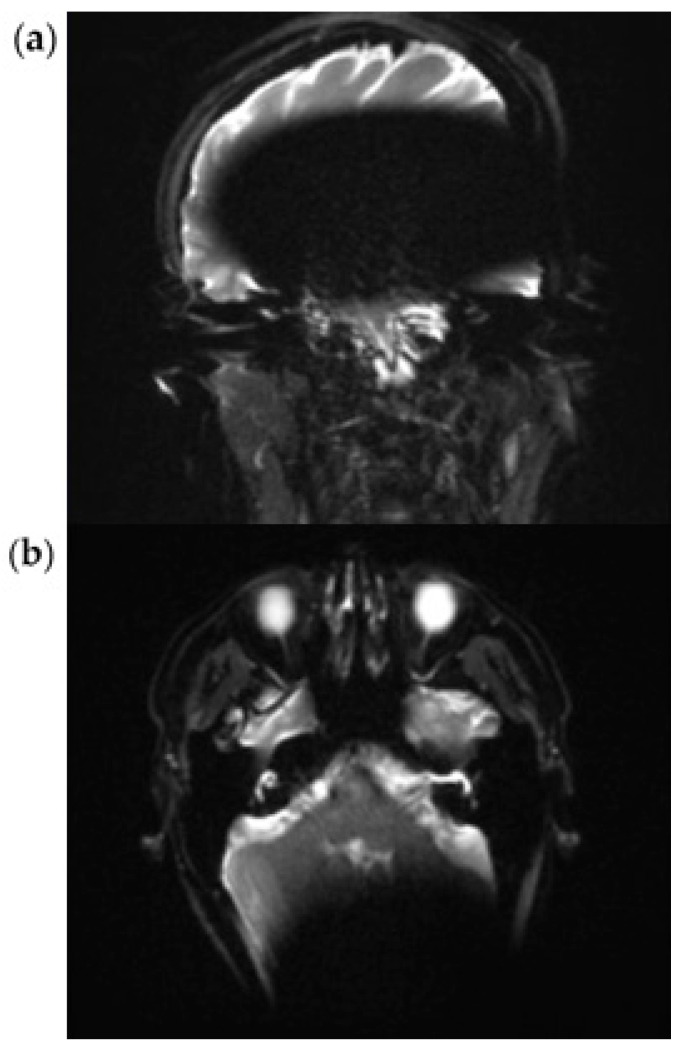
Exemplary scan with a RESOLVE sequence at 3 T in a coronal (**a**) and axial (**b**) plane.

In the axial plane, both the ipsilateral cochlea and the internal auditory canal remain unaffected by the artifact spread. The course of the internal auditory canal can be visualized artifact-free up to the cerebellopontine angle and assessed without any discernible geometric distortions. In the coronal view, however, complete traceability of these structures is not reliably possible due to superimposed artifacts. The same applies to the visualization of the external auditory canal, which can only be assessed to a limited extent in the coronal plane due to artifacts (Figure 3a,b).

### 3.3. HASTE Sequence

In the HASTE sequence (1.5 Tesla), all three subjects exhibited a signal extinction localized around the cochlear implant, consistent with a metal-induced susceptibility artifact. This is localized ipsilaterally, temporoparietally, and occipitally, and manifests as a centrally hypointense area. The mean extent of the artifact in axial orientation is 60 mm (subject 3: 61 mm; subject 4: 48 mm; subject 5: 71 mm). In the coronal plane, the average extension is 102 mm (test subject 3: 128 mm; test subject 4: 125 mm; test subject 5: 54 mm). In cases 3 and 4, the artifact extension in the coronal plane extends beyond the midline. A relevant geometric distortion of the surrounding structures is not recognizable in any of the cases. Minor, unspecific signal inhomogeneities can be seen at the edges of the artifact zones. Due to the image quality, the anatomical detail recognizability is limited in all three subjects.

**Figure 4:**
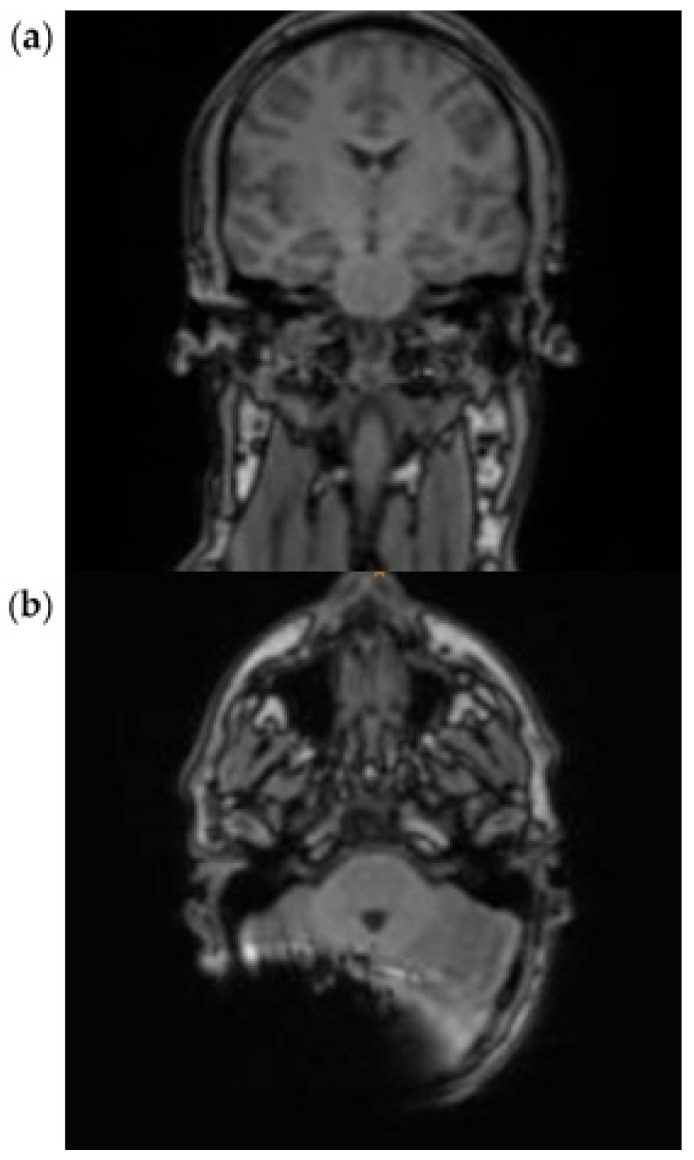
Exemplary scan with HASTE sequence at 1.5 T in a coronal (**a**) and axial (**b**) plane.

In subjects 3, 4, and 5, both the ipsilateral and the contralateral mastoid can be displayed completely and without artifact overlay. The same applies to the internal and external auditory canals, which are imaged artifact-free on both sides (Figure 4a,b).

**Figure 5:**
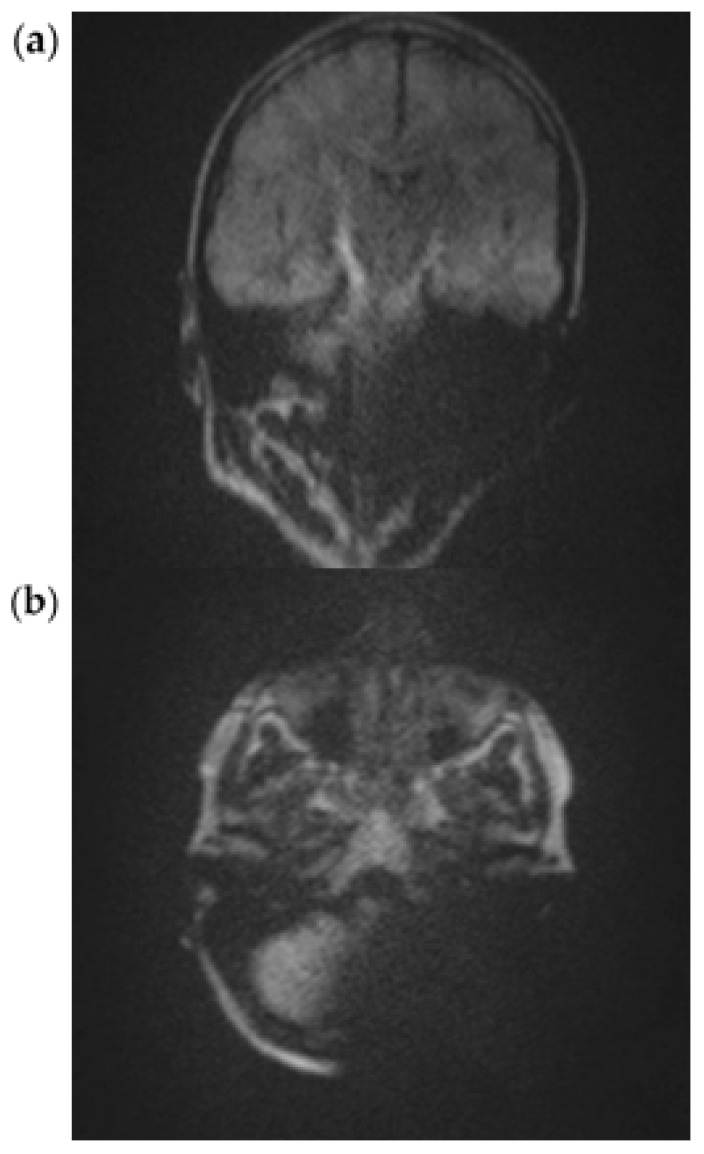
Exemplary scan with HASTE sequence at 1.5 T in a coronal (**a**) and axial (**b**) plane with an EAC distance of 7 cm, without a chin-to-chest position of the head in the scanner.

The effect of a closer position of the implant to the EAC and a non-anteverted head position inside the scanner is shown in Figure 5a,b. Here, the ipsilateral mastoid and internal auditory canal (IAC) are not assessable. For a general overview see Table 2.

SAR values calculated for all used sequences and scanners were between 0.18 and 0.36 (W/kg).

## 4. Discussion

Cholesteatoma assessment by MRI scanning has become a routine procedure in many otologic departments, as it offers an option for a non-surgical second look after cholesteatoma surgery. A high rate of sensitivity and specificity made it a reliable tool [15].

A significant disadvantage of the commonly used epi and non-epi sequences in DWI is susceptibility to magnetic fields and the generation of distortional artifacts [14]. This has, so far, made radiological cholesteatoma follow-up in cochlear implant recipients impossible. The follow-up in cases of subtotal petrocectomy with CI relied on clinical observation and CT scans [19], which has a limited value since CT scans cannot detect cholesteatoma.

Despite this limitation, the rate of cholesteatoma in this group is described to be between 9.3% and 20% [20,21,22].

Subtotal petrosectomy is a reliable surgical technique in cases of COM, Cholesteatoma, temporal bone fractures, otosclerosis, vestibular schwannomas, and inner ear malformations, including a blind sac closure, which makes a visual microscopic observation impossible. Recent publications have reported high rates of recurrent cholesteatoma in patients without cochlear implants after 8 years [23]. The magnetic artifact of the cochlear implant currently makes radiological cholesteatoma follow-up by MRI impossible.

Our findings enable a radiologic assessment of the mastoid with a cochlear implant in place, thereby resolving this issue. The use of RESOLVE or HASTE sequences for cholesteatoma detection makes it possible to assess the mastoid region for the first time. However, it is worth noting that not only is the used sequence important. Additionally, the implant must be placed 12 cm behind the external auditory canal (EAC), and the patient’s head must be positioned inside the MRI scanner in a chin-to-chest position [4]. (Figure 3 and Figure 4). The importance of not solely focusing on the used sequence becomes clear when examining Figure 5. In this case, however, a proper sequence was used; a visualization of the mastoid region was not possible since the implant position and head position were not as recommended. Related to this, the artifact shadowed the region of interest (mastoid).

This proposed guideline needs to be tested in vivo now. Although the HASTE sequence is available at 3 T, we were unable to test it since it was not installed at the time of observation. The shift from an ex vivo position (external with swim cap) to an in vivo (surgical position) might have a minor effect and needs to be considered. A far occipital position of the implant can be assumed to have a negative effect on microphone directionality if using an off-the-ear audioprocessor (AP). Therefore, a behind-the-ear AP could be the AP of choice in these cases.

Although it has been shown that hair cells’ function (residual hearing) is not affected by MRI scanning in cochlear implant recipients [24], specific SAR values are of high importance. The SAR values in our observations, ranging from 0.18 to 0.36 W/kg, were all within the manufacturer’s given limitations: 3.2 W/kg for head MRI (1.5 T) and 1.6 W/kg for head MRI at 3 T (MEDEL, Innsbruck, Austria). Therefore, all scans could be performed even in vivo without any risk to the patient.

## 5. Conclusions

Postoperative cholesteatoma assessment after CI implantation and subtotal petrosectomy appears to be possible under 1.5 T and 3 T, considering the MRI sequence, implant position, and head position.

## Figures and Tables

**Table 1:** Detailed description of the performed sequences.

1.5 T		3 T		
MAGNETOM Altea, Siemens Healthineers, Erlangen, Germany	1.5 T	MAGNETOM Skyra, Siemens Healthineers, Erlangen, Germany	3 T	3 T
Transmit Coil	Body	Transmit Coil	Body	Body
Receive Coil	HeadNeck_20_TCS	Receive Coil	HeadNeck_20	HeadNeck_20
Sequence Type	HASTE DIFFUSION	Sequence Type	EPI DIFFUSION	RESOLVE DIFFUSION
Protocol Name	t2_haste_diff_p2	Protocol Name	ep2d_diff_4scan_trace_p2	resolve_4scan_trace_p2_192
voxel size	1.2 × 1.6 × 3 mm	^3^voxel size	1.2 × 1.2 × 3 mm^3^	1.2 × 1.2 × 3 mm^3^
FOV	230 × 230 mm	^2^FOV	230 × 230 mm^2^	230 × 230 mm^2^
TR	2000 ms	TR	10,400 ms	7280 ms
TE	107 ms	TE	85 ms	65.18 ms
flip angle	150°	flip angle	90°	180°
Slice Thickness	3 mm	Slice Thickness	3 mm	3 mm
Spacing Between Slices	3.3 mm	Spacing Between Slices	3.9 mm	3.9 mm
Acquisition Matrix	192 × 144	Acquisition Matrix	192 × 192	192 × 192
Number of Averages	12	Number of Averages (b0)	2	1
TA	07:17 min	Number of Averages (b1000)	3	1
		TA	03:19 min	04:45 min

**Table 2:** Assessment of the ipsilateral and contralateral mastoid, IAC, and EAC with EPI sequence at 3T, RESOLVE at 3 T, and HASTE at 1.5 T.

Structure	EPI	RESOLVE	HASTE
**Ipsilateral Mastoid**	Not assessable	assessable	assessable
**Contralateral Mastoid**	Not assessable	assessable	assessable
**Ipsilateral IAC**	Not assessable	mainly assessable	assessable
**Contralateral IAC**	Not assessable	assessable	assessable
**Ipsilateral EAC**	Not assessable	mainly assessable	assessable
**Contralateral EAC**	Not assessable	assessable	assessable

## Data Availability

The original contributions presented in the study are included in the article, further inquiries can be directed to the corresponding author.
